# Exploring Erythropoietin and G-CSF Combination Therapy in Chronic Stroke Patients

**DOI:** 10.3390/ijms17040463

**Published:** 2016-03-30

**Authors:** Yoon-Kyum Shin, Sung-Rae Cho

**Affiliations:** 1 Department and Research Institute of Rehabilitation Medicine, Yonsei University College of Medicine, Seoul 03722, Korea; kyum309@hanmail.net; 2 Brain Korea 21 PLUS Project for Medical Science, Yonsei University College of Medicine, Seoul 03722, Korea; 3 Rehabilitation Institute of Neuromuscular Disease, Yonsei University College of Medicine, Seoul 03722, Korea; 4 Yonsei Stem Cell Research Center, Avison Biomedical Research Center, Seoul 03722, Korea

**Keywords:** erythropoietin, granulocyte-colony stimulating factor, combination therapy, stroke, neuroprotection

## Abstract

Erythropoietin (EPO) and granulocyte-colony stimulating factor (G-CSF) are known to have neuroprotective actions. Based on previous reports showing the synergistic effects of EPO+G-CSF combination therapy in experimental models, we investigated the safety of EPO+G-CSF combination therapy in patients with chronic stroke. In a pilot study, 3 patients were treated with EPO and G-CSF for 5 consecutive days, with follow-up on day 30. In an exploratory double-blind study, 6 patients were allocated to treatment with either EPO+G-CSF or placebo. Treatment was applied once a day for 5 days per month over 3 months. Participants were followed up for 6 months. To substantiate safety, vital signs, adverse events, and hematological values were measured on days 0, 5, and 30 in each cycle and on day 180. Functional outcomes were determined on day 0 and 180. In the laboratory measurements, EPO+G-CSF combination therapy significantly elevated erythropoietin, CD34^+^ hematopoietic stem cells, white blood cells, and neutrophils on day 5 of each cycle. There were no observations of serious adverse events. In the functional outcomes, the grip power of the dominant hand was increased in the EPO+G-CSF treatment group. In conclusion, this exploratory study suggests a novel strategy of EPO+G-CSF combination therapy for stroke patients.

## 1. Introduction

Brain injury from ischemic or hemorrhagic stroke results in tissue destruction in the involved brain regions and eventually loss of motor function. Although there have been a variety of efforts to recover motor deficits through training-based interventions, conventional rehabilitation has yielded limited improvements in stroke patients. Therefore, pharmacological treatment using multiple drugs such as erythropoietin (EPO) and granulocyte-colony stimulating factor (G-CSF) has been introduced in clinical trials for stroke patients [1,2,3,4,5,6,7].

Hematopoietic growth factors such as EPO and G-CSF have particularly been utilized to increase circulating blood cells, including red blood cells, white blood cells, and platelets, by stimulating bone marrow production. EPO is commonly known for regulating red blood cell production in patients with anemia [8,9], while G-CSF is generally known for acting on CD34^+^ hematopoietic stem cells to stimulate neutrophil progenitor proliferation and differentiation in patients with neutropenia, as well as mobilizing transplanted bone marrow stem cells in patients with hematological malignancies such as multiple myeloma [10,11].

In addition to the peripheral effects, these hematopoietic factors also stimulate CD34^+^ hematopoietic stem cell migration from the bone marrow into the brain region through peripheral blood circulation [12,13,14]. EPO acts through beneficial mechanisms to exert anti-apoptotic, antioxidant, anti-inflammatory, neural stem cell-facilitating, neurotrophic, and neuroprotective effects [2,15,16]. G-CSF has therapeutic potential by mobilizing blood stem cells in humans, leading to neuroprotective effects and acting against immunotoxicity in mammals [3,12,17,18]. Hematopoietic growth factors help avoid inevitable therapeutic limitations and complications of stem cell transplantation including infection, malignancy, and ethical issues [19]. De La Peña *et al.* [20] also suggested that the co-administration of G-CSF as an adjunctive therapy with umbilical cord blood cell transplantation could overcome the therapeutic limitations of a single therapy by enhancing neuroprotective interactions in animal models.

Ehrenreich *et al.* [1] demonstrated that high-dose intravenous EPO administration can reach the central nervous system, with levels in the cerebrospinal fluid 100 times that of the placebo control, as well as decrease infarct volume after 30 days with no clinical safety issues in patients with acute ischemic stroke. However, Ehrenreich *et al.* [2] found that the death rate of acute stroke patients is related to the interaction between thrombolytic drugs and EPO medication, suggesting that patients treated with thrombolytic drugs should not receive EPO. This result alludes to beneficial outcomes of EPO treatment in ischemic stroke patients with no clinical history of current thrombolytic therapy.

Shyu *et al.* [21] suggested that acute stroke patients who complete five days of G-CSF treatment showed greater improvement of neurological scores, including stroke severity and motor scale, and metabolic activity in the area surrounding the infarction core. Although there were no changes in functional outcome, Sprigg *et al.* [22] found that G-CSF increased CD34^+^ hematopoietic stem cells up to 15-fold at the highest dose compared with placebo, and increased total leukocyte counts up to approximately 4 times compared with pre-treatment, suggesting mobilization of hematopoietic stem cells from the bone marrow into peripheral circulation. Floel *et al.* [23] showed that G-CSF in chronic stroke patients was tolerable and efficacious, with no adverse events and increased leukocyte counts. England *et al.* [24] revealed that G-CSF could raise bone marrow-derived stem cell mobilization by investigating CD34^+^ hematopoietic stem cells fates in the brain of subacute stroke patients, accompanied by the direct effect that given hematopoietic growth factors can cross the blood-brain barrier and act on neurons in the infarct area [17].

Likewise, hematopoietic growth factors are characterized as well-tolerated therapies in patients with stroke [19,25,26,27,28]. Although many studies of promising hematopoietic neuroprotectants have been undertaken for stroke treatment, most trials have focused on acute or subacute stroke treatment with a single administration of either EPO or G-CSF. Previous studies tried to demonstrate the safety and potential beneficial effects of a single pharmacological treatment using either EPO or G-CSF for chronic multiple sclerosis and stroke [23,27,29]. However, these previous reports indicated that further studies are required to clearly demonstrate the safety and feasibility of administering neurotrophic factors for longer time periods or repeated dosing intervals in patients with chronic brain injury.

The present investigation has been inspired by the distinctive and synergistic beneficial neuroprotective effects of EPO and G-CSF in patients with brain injury. Based on the promising results, namely that EPO in combination with G-CSF exerted synergistic effects on tissue plasticity and functional recovery in an experimental stroke model [30], we firstly designed an exploratory study to prepare and expand a double-blind, placebo-controlled trial of EPO+G-CSF combination therapy in chronic stroke patients who had lower risks of treatments than patients in the acute or subacute stage. Hence, we evaluated the safety and synergistic potential of long-term EPO+G-CSF combination therapy with repeated administration in patients with chronic stroke. The aim of this study was to determine the primary safety outcomes of vital signs, adverse events, and laboratory measures, and to substantiate secondary functional outcomes after EPO+G-CSF combination therapy in patients with chronic stroke.

## 2. Results

### 2.1. Participants

**Figure 1:**
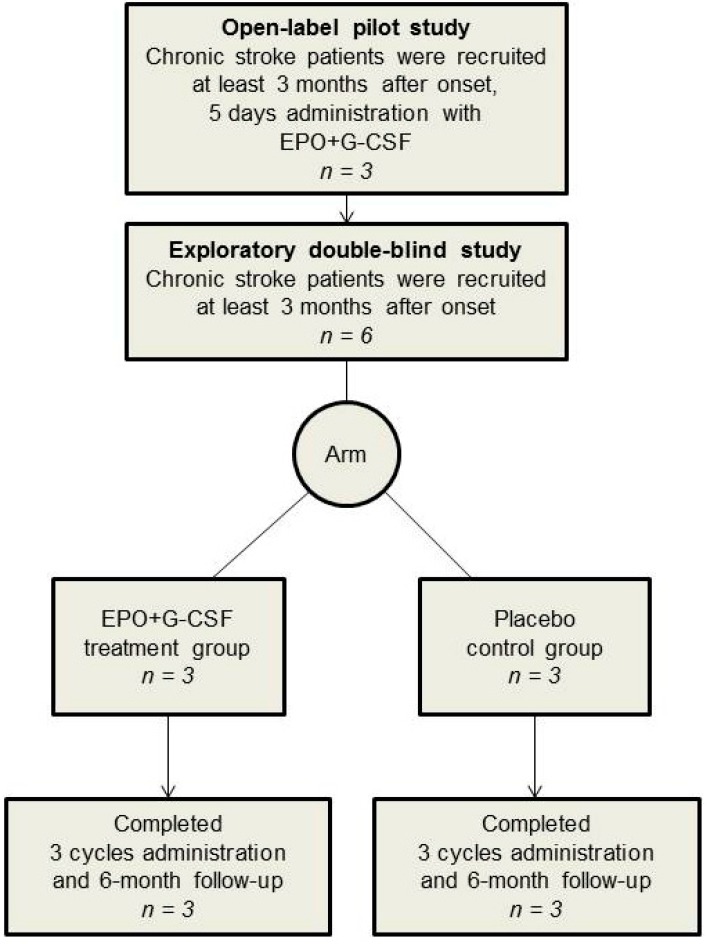
Flow of participants through the study. For a pilot study, three patients with chronic stroke were treated with subcutaneous recombinant human EPO (300 U/kg) and recombinant human G-CSF (10 μg/kg) once a day for 5 consecutive days with follow up on day 30. Six patients with chronic stroke were randomly allocated into two groups (3 EPO+G-CSF and 3 placebo) in an exploratory double-blind study. The EPO+G-CSF arm was administered as the same treatment as the pilot study per cycle (one month) over a total of 3 cycles. The placebo arm had the same schedule of subcutaneous saline injections as the treatment arm. After completing three treatment cycles, participants in the double-blind study received follow-up for six months from the start of combination therapy in the same clinic.

Three participants who received EPO+G-CSF treatment were included in an open-label pilot study. A total of six participants were included in an exploratory double-blinded study. Two arms (3 EPO+G-CSF and 3 placebo control) were matched at baseline (Figure 1 and Table 1). All participants completed three experimental cycles and the 6-month follow-up evaluation in an exploratory double-blind study.

### 2.2. Laboratory Measurements

**Figure 2:**
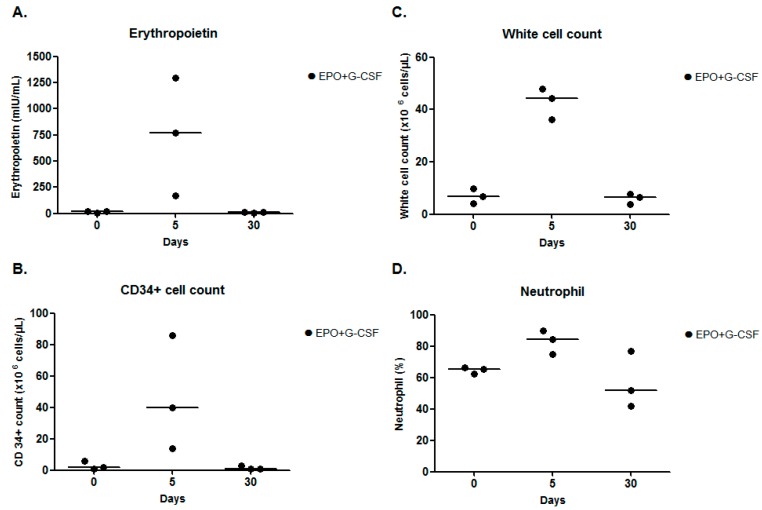
Laboratory measurements for a pilot study. (**A**) Erythropoietin concentration; (**B**) CD34^+^ cell; (**C**) white cell count; and (**D**) neutrophil on days 0, 5, and 30 of EPO+G-CSF combination therapy. EPO+G-CSF treatment showed peaks on four parameters on day 5. The hematological patterns returned to baseline values on day 30. The line was presented as a median value.

In the pilot study, compared to pre-treatment, EPO+G-CSF combination therapy (*n* = 3) showed increases of erythropoietin concentrations (767.00 (interquartile range; 469.00–1032.00) *versus* 16.80 (10.74–16.95) mIU/mL), CD34^+^ hematopoietic stem cells (40.00 (27.00–63.00) *versus* 2.00 (1.50–4.00) cells/μL), white blood cells (44.36 (40.27–46.16) *versus* 6.91 (5.48–8.36) cells/μL), and neutrophils (84.20 (79.60–86.90) *versus* 65.40 (63.75–65.85) %) on day 5. The peaks of these four parameters had returned to baseline on day 30 (Figure 2A–D and Table 2).

**Figure 3:**
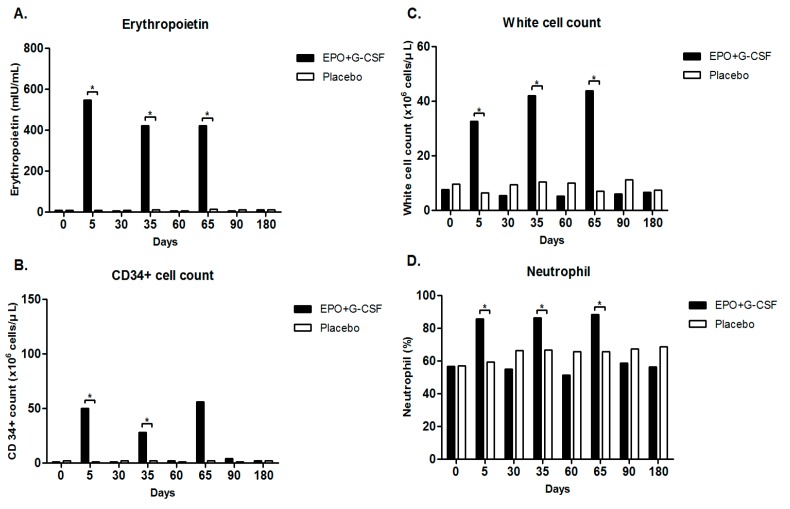
Laboratory measures for a total of 3 cycles in an exploratory double-blind study. (**A**) Erythropoietin concentration; (**B**) CD34^+^ cell; (**C**) white cell count; and (**D**) neutrophil on days 0, 5, and 30 of each cycle, and 6-month by EPO+G-CSF combination therapy or placebo control. EPO+G-CSF group showed significant peaks on four parameters on day 5 compared with placebo control in each cycle. The hematological patterns repeated every three cycles and returned to baseline values in participants who completed 3 cycles of intervention. Values are median. * *p* < 0.05 by Mann-Whitney *U* test.

In an exploratory double-blind study, compared to placebo treatment (*n* = 3), EPO+G-CSF combination therapy (*n* = 3) significantly elevated erythropoietin concentrations (547.00 (419.50–601.00) *versus* 8.89 (7.46–13.35) mIU/mL, *p* = 0.049) by Mann-Whitney *U* test, CD34^+^ hematopoietic stem cells (50.00 (33.00–61.00) *versus* 1.00 (1.00–3.00) cells/μL, *p* = 0.046), white blood cells (32.56 (29.59–45.68) *versus* 6.41 (5.93–7.44) cells/μL, *p* = 0.049), and neutrophils (85.80 (85.40–86.75) *versus* 59.30 (55.85–60.50)%, *p* = 0.049) on day 5 (Figure 3A–D and Table 3). EPO+G-CSF treatment also showed a significant increase in reticulocyte counts and immature red blood cells (92.90 (82.40–95.50) *versus* 48.80 (44.45–52.75) cells/μL, *p* = 0.049) on day 5 (Table 3). On the other hand, EPO+G-CSF treatment did not change red blood cell counts, hematocrit, or hemoglobin values (Table 3). Hematological values returned to baseline on day 30. These hematological patterns repeated every three cycles and returned to baseline values in participants who completed 3 cycles of intervention (Figure 3A–D and Table 3).

### 2.3. Clinical Outcomes

Mild to severe adverse events were monitored in all participants. A total of 20 side effects were considered using a checklist (Table 4). In the open-label pilot study, all participants who received EPO+G-CSF suffered from mild lower back pain, two of three participants suffered from headache, and one of three participants showed intermittent vomiting during the 5 consecutive days of treatment.

In an exploratory double-blind study, one of 3 participants who received EPO+G-CSF suffered from mild lower back pain during the 5 consecutive days of each treatment period (Table 4). However, there were no observations of serious adverse events such as cardiovascular complications, infections, or mortality. No significant deviations from the normal range were found in vital signs including systolic and diastolic blood pressure, pulse rate, and body temperature after EPO+G-CSF combination therapy or placebo treatment. No participants had evidence of pneumonia or heart problems in chest X-rays and electrocardiograms up to 6 months after treatment.

In the pre-treatment evaluation of cognitive and functional levels, there were no differences between the EPO+G-CSF and placebo groups in mini-mental status examination (MMSE) and modified Barthel index (MBI). This result suggested that initial conditions of groups were well matched. Six months after treatment, no significant differences had emerged with respect to MMSE and MBI between groups. Grip and pinch power (tip, lateral, and palmar pinch) and hand dexterity evaluated by the box-and-block test in the affected hand did not show significant differences between groups (Table 5). However, the grip power of the dominant hand was significantly increased in the EPO+G-CSF treatment group compared to placebo control (46.00 (40.00–49.00) *versus* 39.00 (33.35–39.50) kg, *p* = 0.049 by Mann-Whitney *U* test) (Table 5).

## 3. Discussion

This study is the first exploratory double-blind study based on the pilot study to evaluate long-term EPO+G-CSF combination therapy with repetitive administration in patients with chronic stroke. Although EPO and G-CSF have been commonly used individually as hematopoietic growth factors in the treatment of anemia and neutropenia, these pharmacological agents have been recently reported to have neuroprotective and regenerative potential as neurotrophic factors in stroke [27,31,32]. Therefore, we examined a novel approach of administering EPO and G-CSF concurrently, which has been known to exert synergistic effects in promoting angiogenesis, neurogenesis and functional recovery in experimental stroke models [30,33]. Although combined administration of EPO and G-CSF represents a promising therapeutic strategy for stroke patients, most studies have focused on peripheral efficacy for the treatment of myelodysplastic syndromes [34,35,36,37]. Based on these previous reports showing the therapeutic advantages of an EPO and G-CSF combination [30,33], we evaluated whether EPO+G-CSF combination therapy safely yielded hematological and functional improvement in stroke patients.

We found that repetitive and long-term combined use of EPO and G-CSF was well tolerated and not associated with serious adverse events, supporting the safety of dual administration in stroke patients. Although serious concerns remain for cardiovascular and hematopoietic events based on the use of hematopoietic growth factors due to increased production of peripheral blood cells, no complications leading to thromboembolic accidents or aggravation leading to mortality occurred in this study [1,2,23,24,38]. Rather, mild adverse events such as musculoskeletal pain and headache were observed during the five consecutive days of each treatment period.

In the laboratory measurements, we found that EPO+G-CSF treatment significantly elevated erythropoietin, CD34^+^ hematopoietic stem cells, white blood cells, and neutrophils in the peripheral blood in patients with chronic stroke. All participants who received EPO+G-CSF achieved the peak values on day 5 in each cycle, followed by a hematological trend to return toward baseline within 30 days after drug administration. Despite significant increases of white blood cells and neutrophils, the EPO+G-CSF group did not show significant complications of vascular events such as thromboembolic accidents that might worsen primary stroke symptoms, consistent with previous reports [19,23]. While reticulocyte levels increased, red blood cells, hematocrit and hemoglobin did not increase in accordance with other studies [1,4]. An increase of hematocrit carries the potential risk of high blood pressure followed by thromboembolic events and further secondary ischemic injury [39]. However, our study demonstrated that blood pressure did not deviate from the normal range after EPO+G-CSF combination therapy. The hematological responses during EPO+G-CSF treatment focused on the safety of combined administration of EPO and G-CSF as neuroprotective agents.

In the functional outcomes of MMSE and MBI, no statistically significant difference was found between the EPO+G-CSF and placebo groups. It might be possible that chronic stroke participants, who showed no cognitive impairment and ADL with minimal assist, represent a ceiling effect to the combination therapy since they already have relatively higher functional level in the initial assessment. Although no significant differences between groups were seen with respect to functional improvement on power and dexterity in the affected hand, there was a significant improvement in the grip power of the dominant hand 6 months after EPO+G-CSF combination therapy.

Previous studies indicate that hematopoietic growth factors encourage functional improvement in disability, dependence, and cognitive functions in patients with acute stroke or coronary artery disease [21,38]. Because our participants were in a chronic stage, we might have expected to observe an improvement of fine motor functions rather than gross motor functions involved in daily activities [23,24]. The present clinical trial of EPO+G-CSF combination therapy should be interpreted with caution due to the limited sample size. Small numbers of subjects in this study were not gender and age matched, resulting in reduced statistical power. The nature of our longitudinal study with serial intervention per cycle (one month) over a total of 3 cycles and six months follow-up during a whole procedure, causing inconvenient participation, may represent limitations of our study. As another limitation, we missed d-mannitol as a vehicle of G-CSF in the placebo control. Although we should have considered two vehicles (saline and d-mannitol), d-mannitol did not affect the double-blind test because its color and agent form were the same as normal saline, as colorless liquid.

## 4. Materials and Methods

### 4.1. Study Design and Ethics

We performed a pilot study followed by an exploratory double-blind study of EPO+G-CSF combination therapy in patients with chronic stroke. We conducted this trial in accordance with the Declaration of Helsinki and Good Clinical Practices. The present study was approved by Institutional Review Board of Severance Hospital, Korea Food and Drug Administration (4-2010-0468), and registered on an international registry system, ClinicalTrials.gov (NCT02018406).

### 4.2. Participant Screening and Enrollment

After obtaining signed informed consent, patients were screened using medical history, a physical assessment, routine blood laboratory tests, and brain magnetic resonance imaging (MRI) findings to confirm study eligibility.

Inclusion criteria were as follows: (1) over 20 years old; (2) voluntary participants; and (3) at least 3 months after onset of ischemic or hemorrhagic stroke. Exclusion criteria were as follows: (1) under 20 years old; (2) participants who could not voluntarily consent; (3) encephalopathy from brain tumor and infection; (4) current thrombolytic therapy including warfarin or coumadin medications; (5) leukopenia, thrombocytopenia, or polycythemia; (6) malignant diseases, malignant hypertension, myeloproliferative disorder, septic embolism, or hyperkalemia; (7) hepatic or renal dysfunction with serum creatinine >3 mg/dL; (8) allergic reactions to exogenous EPO or G-CSF; (9) possessing exclusion criteria for MRI test; (10) pregnant or breast feeding in women; (11) body temperature over 38 °C; (12) blood pressure over 140/90 mmHg at pre-treatment; (13) blood pressure over 160/100 mm Hg during intervention; (14) hemoglobin > 15 g/dL at pre-treatment; (15) hemoglobin > 17 g/dL during intervention; (16) pneumonia detected by chest X-ray; and (17) recurrent history of aspiration pneumonia.

In an open-label pilot study, participants consented to be treated with EPO+G-CSF for 5 consecutive days, and be followed up on day 30. In an exploratory double-blind study, following enrollment of eligible participants, randomization was carried out in an institutional clinical pharmacy. A clinical pharmacist unfamiliar with participants and investigators used a table of random sampling numbers to randomly allocate participants to either the placebo or EPO+G-CSF arm.

### 4.3. Drug Administration

Three patients with chronic stroke participated in a pilot study. They were treated with subcutaneous recombinant human EPO (300 U/kg, CJ HealthCare Corp., Seoul, Korea) in the sodium chloride solution (10,000 U/mL) and recombinant human G-CSF (10 μg/kg, CJ HealthCare Corp., Seoul, Korea) in the d-mannitol solution (250 μg/mL) separately at the same time, once a day for 5 consecutive days, with follow-up on day 30.

Six patients with chronic stroke (3 EPO+G-CSF and 3 placebo) were randomly allocated into two groups in an exploratory double-blind study. The EPO+G-CSF arm were administered as the same treatment as the pilot study per cycle (one month) over a total of 3 cycles. The placebo arm had the same schedule and volume of subcutaneous saline injections as the treatment arm. These doses were in line with the published safety data [1,23,24].

After completing three treatment cycles, participants were followed up for six months from the start of combination therapy in the same clinic. To substantiate safety, vital signs, adverse events, and hematological tests were recorded at each follow-up visit in all patients. To measure functional outcomes, MMSE, MBI, grip and pinch power tests and box-and-block tests were performed on days 0 and 180. All patients completed a six-month follow-up (Figure 1).

### 4.4. Laboratory Measures

Hematological examinations to measure erythropoietin concentration, CD34^+^ hematopoietic stem cells, white blood cells including neutrophils, lymphocytes, and monocytes, red blood cells, reticulocytes, hemoglobin, hematocrit, glucose, creatinine, and C-reactive protein were conducted on days 0, 5, 30 in each cycle, and 6 months after treatment commenced. Blood samples were obtained via venous puncture by medical laboratory scientists, and analyzed according to the institutional standard guidelines as follows. (1) Serum EPO concentration was measured by an enzyme-labeled chemiluminescent immunometric assay using an immunoassay system (Immulite 2000 XPi, Siemens, NY, USA); (2) CD34^+^ cell count was performed using a flow cytometer and software (Navios, Beckman Coulter, Villepinte, France); (3) Total white blood cells and differentiated subtypes (neutrophils, lymphocytes, and monocytes), red blood cells, reticulocytes, hemoglobin, hematocrit were measured using automated blood cell counters (ADVIA 2120i, Siemens, NY, USA; XN-10, XN-20, Sysmex, Kobe, Japan); (4) Glucose was measured by the hexokinase method using a commercial kits with an automated chemistry analyzer (Hitachi 7600-200-DDP, Hitachi Ltd., Tokyo, Japan); (5) Creatinine was measured by the Jaffe method using a commercial kit with an automated chemistry analyzer (Hitachi 7600-210-DDP, Hitachi Ltd., Tokyo, Japan); (6) C-reactive protein was measured by the turbidimetric latex agglutination method, using a commercial kit with an automated biochemical analyzer (Hitachi 7600 P module, Hitachi Ltd., Tokyo, Japan).

### 4.5. Clinical Outcomes

Vital signs (blood pressure, heart rate, and body temperature) and adverse events were evaluated on the 5 consecutive treatment days, day 30 in each cycle, and 6 months after treatment commenced. Vital signs were measured by nurses using an automatic blood pressure monitor (TM-2655, A&D Co., Ltd., Tokyo, Japan) and an digital thermometer (DT-502EC, A&D Co., Ltd., Tokyo, Japan). Chest X-rays (KXO-80XM, TOSHIBA, Tokyo, Japan) and electrocardiograms (MAC 5000, GE Marquette Medical Systems, Milwaukee, WI, USA) were evaluated to screen and exclude pneumonia and heart failure on days 0 and 180. Functional outcome measures including MMSE, MBI, grip and pinch power, and the box-and-block test for hand dexterity were evaluated on days 0 and 180. MMSE evaluates cognitive functions such as registration, attention, calculation, recall, language, ability to follow simple commands, and orientation. MMSE scores range from 0 to 30, with a lower score indicating cognitive impairment and the highest score indicating no cognitive impairment [40]. MBI contains 10 items of activities in daily living (ADL) such as feeding, grooming, dressing, bathing, toilet use, transfer, mobility, stair climbing, bowel control, and bladder control. MBI scores range from 0 to 100, with a lower score indicating impediment and a higher score indicating independence [41]. Grip and pinch power of both hands was measured using a Jamar hand-held dynamometer (PC 5030J1, Preston Co., Carson City, NV, USA) and a Jamar hydraulic pinch gauge (PC 5030HPG, Preston Co., Carson City, NV, USA). The maximum gauge of each participant was recorded [42]. The box-and-block test was performed using two adjacent boxes over a partition. One of them was filled with 150 cube-shaped blocks (2.5 cm^3^). A participant moved blocks from one box to the other. The number of blocks moved within 60 seconds was counted by occupational therapists [43]. All medical information and clinical measures for each participant were evaluated by medical laboratory scientists, nurses, radiological technicians and occupational therapists blinded to the trial.

### 4.6. Statistical Analysis

All statistical analyses were performed with SPSS Statistics 23 (IBM Corp., Armonk, NY, USA). Statistical analyses were performed after all participants completed experimental procedures. Median and interquartile ranges (25th–75th percentiles) were used for the non-normally distributed variables. Mann-Whitney *U* tests were used to compare groups for treatment effects. Statistical significance was defined as *p* < 0.05.

## 5. Conclusions

This study provides a novel strategy of EPO+G-CSF combination therapy for stroke patients. Based on the safety outcomes of EPO+G-CSF combination therapy in chronic stroke patients, further clinical trials are required to expand the sample size of chronic stroke patients and inclusion criteria including acute or subacute stroke patients to test the efficacy of EPO+G-CSF combination therapy. Therefore, the design of our further study will be considered as a single cycle in large numbers of stroke patients as a neuroprotective strategy. Ultimately, it will provide a definite therapeutic rationale by showing the utility of this combination therapy in a large-sample-size study. Taken together, this exploratory double-blind study after a pilot study provides the first evidence that EPO+G-CSF may have safe and beneficial therapeutic potential for chronic stroke patients.

## Figures and Tables

**Table 1:** Clinical characteristics of patients at time of enrollment in the study.

Characteristics	Open-Label Pilot Study	Exploratory Double-Blind Study	
	EPO+G-CSF (*n* = 3)	EPO+G-CSF (*n* = 3)	Placebo (*n* = 3)
Age (years; range)	30.00 (25.00–32.00)	45.00 (44.00–45.00)	34.00 (31.50–34.50)
Sex (male/female)	3/0	3/0	2/1
Height (cm)	172.00 (166.50–173.50)	169.00 (167.00–172.00)	170.00 (167.50–172.00)
Weight (kg)	50.60 (49.95–64.30)	65.00 (57.50–72.15)	79.80 (71.40–82.15)
Body Mass Index (kg/m^2^)	19.50 (18.10–22.50)	22.80 (20.60–24.35)	27.60 (25.35–27.75)
Etiology (ischemic/hemorrhagic)	2/1	1/2	2/1
Damaged Region (BG ^†^/F-P ^‡^/CR ^§^)	2/1/0	2/1/0	2/0/1
Modified Barthel Index	57.00 (57.00–75.00)	93.00 (82.50–94.00)	96.00 (90.50–98.00)
Mini-Mental Status Examination	29.00 (20.50–29.50)	25.00 (24.50–26.50)	30.00 (30.00–30.00)
SBP ^¶^ (mmHg)	135.00 (135.00–135.50)	134.00 (128.00–139.50)	135.00 (128.00–136.50)
DBP ** (mmHg)	82.00 (79.50–84.50)	82.00 (76.50–82.00)	87.00 (82.50–90.00)
Heart Rate (pulse/min)	80.00 (75.00–85.50)	64.00 (61.50–83.00)	79.00 (74.50–82.50)
Body Temperature (°)	37.00 (36.85–37.15)	36.50 (36.50–36.65)	36.70 (36.70–36.75)
Adverse Effects (number)			
Back Pain	3	1	0
Headache	2	0	1
Vomiting	1	0	0

Values are mean ± SE; ^†^ BG: basal ganglia; ^‡^ F-P: fronto-parietal cortex; ^§^ CR: corona radiata; ^¶^ SBP: systolic blood pressure; ** DBP: diastolic blood pressure.

**Table 2:** Hematological parameters of the EPO and G-CSF combination therapy in stroke patients in a pilot study.

EPO+G-CSF (*n* = 3)				
Parameter	Normal Range	Pre-Treatment	Day 5	Day 30
Glucose (mg/dL)	70–110	96.00 (90.50–103.50)	86.00 (81.50–89.00)	86.00 (83.00–92.50)
Creatinine (mg/dL)	0.49–1.19	0.89 (0.77–1.06)	0.92 (0.85–1.03)	0.74 (0.70–0.96)
CRP ^†^ (mg/L)	0–8	2.85 (1.43–9.76)	3.03 (2.79–4.08)	2.84 (1.42–5.90)
Reti count ^‡^ (cells/μL)	20.8–109.6	53.80 (50.90–69.95)	76.40 (71.15–88.85)	41.50 (40.95–49.80)
RBC ^§^ (cells/μL)	4.5–6.1 × 10^6^	4.69 (4.37–4.87)	4.94 (4.73–5.18)	4.85 (4.74–5.08)
Hemoglobin (g/dL)	13–17	15.00 (14.45–15.15)	14.20 (14.15–14.35)	14.30 (13.80–14.35)
Hematocrit (%)	40–52	43.00 (42.95–43.05)	43.00 (42.45–43.45)	43.50 (43.05–43.60)
WBC ^¶^ (cells/μL)	4–10.8 × 10^3^	6.91 (5.48–8.36)	44.36 (40.27–46.16)	6.39 (5.14–6.98)
Neutrophil (%)	20–70	65.40 (63.75–65.85)	84.20 (79.60–86.90)	51.80 (46.90–64.40)
Lymphocyte (%)	15–40	26.80 (25.75–27.10)	9.50 (6.95–10.25)	34.20 (24.55–40.70)
Monocyte (%)	2–8	5.90 (5.50–6.35)	4.80 (4.45–5.90)	6.50 (6.25–7.50)
EPO ** (mIU/mL)	3.5–16.2	16.80 (10.74–16.95)	767.00 (469.00–1032.00)	9.24 (7.09–12.32)
CD34^+^ (cells/μL)	-	2.00 (1.50–4.00)	40.00 (27.00–63.00)	1.00 (1.00–2.00)

Values are median (interquartile range); ^†^ CRP: C-reactive protein; ^‡^ Reti count: Reticulocyte count; ^§^ RBC: red blood cell; ^¶^ WBC: white blood cell; ** EPO: erythropoietin.

**Table 3:** Hematological parameters of the EPO and G-CSF combination therapy in stroke patients in an exploratory double-blind study.

**EPO+G-CSF (*n* = 3)**									
**Parameter**	**Normal Range**	**Day 0**	**Day 5**	**Day 30**	**Day 35**	**Day 60**	**Day 65**	**Day 90**	**Day 180**
Glucose (mg/dL)	70–110	89.00 (82.50–93.50)	108.00 (97.00–113.00)	90.00 (90.00–92.50)	80.00 (77.00–88.00)	90.00 (86.50–135.00)	113.00 (106.00–125.00)	100.00 (96.50–103.50)	107.00 (105.00–122.00) *
Creatinine (mg/dL)	0.49–1.19	1.06 (0.89–1.13)	1.09 (0.94–1.17)	0.95 (0.86–1.08)	1.05 (0.92–1.10)	1.07 (0.96–1.11)	1.23 (0.99–1.24)	0.95 (0.87–1.07)	1.17 (0.97–1.22)
CRP ^†^ (mg/L)	0–8	0.40 (0.20–2.00)	8.20 (6.84–13.70)	0.50 (0.25–0.50)	10.70 (6.99–12.40)	0.60 (0.30–0.60)	5.40 (5.33–13.55) *	0.60 (0.30–0.85)	1.40 (0.95–1.49)
Reti count ^‡^ (cells/μL)	20.8–109.6	61.20 (56.00–65.45)	92.90 (82.40–95.50) *	31.10 (26.95–41.30)	82.00 (74.80–82.15)	20.00 (18.85–26.10)	58.50 (47.35–70.90)	25.20 (24.50–30.60)	81.20 (73.80–86.60)
RBC ^§^ (cells/μL)	(4.5–6.1) × 10^6^	4.18 (4.11–4.44)	4.20 (4.18–4.35)	4.60 (4.37–4.81)	4.67 (4.61–4.83)	4.64 (4.60–4.66)	4.81 (4.75–5.01)	4.94 (4.91–5.04)	4.32 (4.24–4.58)
Hemoglobin (g/dL)	13–17	12.80 (12.40–13.65)	12.70 (12.60–13.35)	14.20 (13.25–15.05)	14.50 (14.20–15.00)	14.00 (13.70–14.15)	14.70 (14.35–15.40)	14.90 (14.45–15.10)	13.20 (12.90–14.10)
Hematocrit (%)	40–52	38.20 (36.80–41.15)	38.10 (37.95–40.70)	42.70 (40.00–45.25)	42.80 (42.45–45.30)	42.00 (41.85–43.00)	44.20 (44.05–46.75)	46.20 (44.40–46.25)	38.90 (37.95–41.25)
WBC ^¶^ (cells/μL)	(4–10.8) × 10^3^	7.52 (6.15–7.53)	32.56 (29.59–45.68) *	5.39 (4.99–6.71)	41.96 (39.51–48.73) *	5.17 (4.69–5.65)	43.79 (37.16–49.95) *	5.98 (5.73–7.50)	6.55 (5.71–7.44)
Neutrophil (%)	20–70	56.50 (55.70–60.55)	85.80 (85.40–86.75) *	55.00 (44.55–62.85)	86.40 (85.10–87.20) *	51.20 (47.55–52.95)	88.40 (85.30–89.50) *	58.70 (51.85–63.55)	56.20 (53.10–60.95)
Lymphocyte (%)	15–40	31.10 (29.15–33.50)	6.90 (6.80–8.45) *	30.40 (26.50–41.65)	6.80 (6.20–8.60) *	35.50 (33.30–41.15)	7.10 (6.30–9.80) *	25.90 (25.85–35.15)	29.50 (27.50–35.15)
Monocyte (%)	2–8	5.80 (5.05–6.75)	4.90 (3.90–4.95)	6.10 (5.45–6.40)	4.20 (3.45–4.25)	5.70 (4.80–6.25)	2.40 (2.35–3.10) *	4.70 (4.15–7.10)	6.90 (6.05–7.10)
EPO ** (mIU/mL)	3.5–16.2	7.30 (5.65–9.40)	547.00 (419.50–601.00) *	4.28 (3.64–6.89)	421.00 (325.00–570.00) *	5.30 (4.40–7.46)	420.00 (361.00–590.00) *	5.20 (4.10–6.15) *	10.60 (9.65–11.75)
CD34^+^ (cells/μL)	-	1.00 (0.50–2.50)	50.00 (33.00–61.00) *	1.00 (1.00–3.50)	28.00 (18.00–78.50) *	2.00 (1.50–4.00)	56.00 (30.00–73.00)	4.00 (2.50–4.00)	2.00 (2.00–2.50)
**Placebo (*n* = 3)**									
**Parameter**	**Normal Range**	**Day 0**	**Day 5**	**Day 30**	**Day 35**	**Day 60**	**Day 65**	**Day 90**	**Day 180**
Glucose (mg/dL)	70–110	87.00 (84.50–89.50)	100.00 (95.50–122.00)	99.00 (94.00–99.50)	90.00 (85.50–97.00)	95.00 (94.50–106.50)	89.00 (88.50–95.50)	98.00 (95.50–98.00)	90.00 (88.00–94.00)
Creatinine (mg/dL)	0.49–1.19	1.09 (1.02–1.12)	1.04 (0.99–1.12)	1.12 (1.02–1.15)	1.13 (0.98–1.14)	1.13 (1.01–1.22)	1.07 (0.91–1.25)	1.14 (1.02–1.15)	1.02 (1.00–1.08)
CRP ^†^ (mg/L)	0–8	0.00 (0.00–0.00)	1.65 (0.83–16.25)	0.50 (0.25–0.94)	0.50 (0.25–1.04)	0.50 (0.25–0.79)	0.50 (0.25–1.24)	2.02 (1.01–2.05)	0.44 (0.22–1.26)
Reti count ^‡^ (cells/μL)	20.8–109.6	84.10 (71.60–97.05)	48.80 (44.45–52.75)	53.20 (52.15–95.95)	80.00 (54.00–85.10)	89.10 (69.00–107.95)	58.80 (58.00–101.75)	82.70 (58.45–86.45)	74.50 (68.75–82.35)
RBC ^§^ (cells/μL)	(4.5–6.1) × 10^6^	5.02 (4.55–5.14)	4.89 (4.44–4.96)	5.09 (4.66–5.23)	4.49 (4.39–4.82)	5.08 (4.74–5.26)	4.83 (4.51–5.06)	4.73 (4.59–5.26)	5.10 (4.71–5.45)
Hemoglobin (g/dL)	13–17	14.60 (13.55–15.00)	14.80 (13.35–14.85)	15.40 (14.10–15.45)	13.80 (13.35–14.55)	15.10 (14.25–15.45)	14.60 (13.65–15.35)	14.30 (13.85–16.10)	15.00 (13.95–16.45)
Hematocrit (%)	40–52	44.00 (39.95–44.15)	42.50 (38.85–42.75)	43.70 (40.55–44.90)	38.40 (38.30–41.60)	44.00 (41.40–45.20)	41.80 (39.35–43.85)	40.60 (40.15–45.55)	43.80 (40.95–47.15)
WBC ^¶^ (cells/μL)	(4–10.8) × 10^3^	9.57 (7.63–9.60)	6.41 (5.93–7.44)	9.33 (8.29–10.23)	10.48 (8.11–10.58)	10.06 (8.01–10.45)	7.01 (6.53–8.49)	11.11 (7.66–11.20)	7.32 (6.53–9.22)
Neutrophil (%)	20–70	56.90 (56.45–60.50)	59.30 (55.85–60.50)	66.40 (60.50–71.85)	66.60 (59.50–69.90)	65.70 (60.05–68.90)	65.60 (55.40–73.25)	67.40 (58.80–69.55)	67.40 (56.65–68.10)
Lymphocyte (%)	15–40	30.70 (28.25–31.55)	29.70 (28.60–32.25)	24.70 (20.36–30.30)	23.70 (20.95–27.40)	26.10 (23.30–28.30)	23.80 (22.25–30.90)	24.10 (21.65–29.45)	24.10 (23.00–30.60)
Monocyte (%)	2–8	6.30 (5.55–6.30)	6.60 (5.40–6.80)	4.40 (4.30–4.85)	4.90 (3.95–5.30)	4.60 (4.60–4.70)	5.30 (5.20–6.05)	5.40 (5.35–5.90)	5.30 (4.90–5.55)
EPO ** (mIU/mL)	3.5–16.2	7.93 (7.52–9.42)	8.89 (7.46–13.35)	8.65 (7.36–9.93)	11.10 (10.41–12.55)	6.60 (3.72–10.70)	12.90 (11.15–13.25)	11.80 (11.60–13.80)	11.40 (10.95–12.10)
CD34^+^ (cells/μL)	-	2.00 (2.00–3.00)	1.00 (1.00–3.00)	2.00 (1.50–4.50)	2.00 (1.50–4.00)	1.00 (1.00–4.00)	2.00 (1.50–5.50)	1.00 (1.00–3.50)	2.00 (1.50–4.00)

Values are median (interquartile range); * *p* < 0.05; ^†^ CRP: C-reactive protein; ^‡^ Reti count: Reticulocyte count; ^§^ RBC: red blood cell; ^¶^ WBC: white blood cell; ** EPO: erythropoietin.

**Table 4:** Adverse events from EPO+G-CSF administration in all participants.

Adverse Effects (Number)	Open-Label Pilot Study	Exploratory Double-Blind Study	
	EPO+G-CSF (*n* = 3)	EPO+G-CSF (*n* = 3)	Placebo (*n* = 3)
Shock	0	0	0
Seizure	0	0	0
Dyspnea	0	0	0
High blood pressure	0	0	0
Hemorrhagic accident	0	0	0
Myocardial/cerebral infarction	0	0	0
Jaundice	0	0	0
Allergy	0	0	0
Itching and rash	0	0	0
Stomachache	0	0	0
Dyspepsia	0	0	0
Vomiting	1	0	0
Diarrhea	0	0	0
Headache	2	0	1
Dizziness	0	0	0
Fever	0	0	0
Chill	0	0	0
Insomnia	0	0	0
Fatigue	0	0	0
Skeletal muscle pain	3	1	0

Values are number of subjects.

**Table 5:** Hand function outcomes of the EPO and G-CSF combination therapy in an exploratory double-blind study.

Hand Side	Evaluation Contents	Normal Range	Day 0		Day 180	
			EPO+G-CSF (*n* = 3)	Placebo (*n* = 3)	EPO+G-CSF (*n* = 3)	Placebo (*n* = 3)
Affected Hand	Grip Power (kg)	46.9–48.3	0.00 (0.00–17.00)	1.00 (0.50–23.50)	0.00 (0.00–22.00)	4.00 (2.00–16.50)
	Tip Pinch Power (kg)	5.1–6.7	0.00 (0.00–2.25)	0.00 (0.00–2.00)	0.00 (0.00–2.75)	0.00 (0.00–2.00)
	Lateral Pinch Power (kg)	7.6–9.5	0.00 (0.00–4.25)	2.50 (1.25–6.75)	0.00 (0.00–5.25)	2.00 (1.00–3.50)
	Palmar Pinch Power (kg)	9.3–9.8	0.00 (0.00–3.75)	0.00 (0.00–2.50)	0.00 (0.00–4.50)	0.00 (0.00–2.50)
	Box and Block (numbers)	81–83	0.00 (0.00–29.00)	0.00 (0.00–25.00)	0.00 (0.00–37.00)	12.00 (6.00–32.00)
Dominant Hand	Grip Power (kg)	46.9–48.3	42.00 (39.00–47.00)	38.00 (35.00–43.00)	46.00 (44.00–49.00) *	39.00 (33.50–39.50)
	Tip Pinch Power (kg)	5.1–6.7	7.50 (6.75–8.00)	4.50 (4.00–5.50)	7.50 (6.50–8.25)	4.50 (4.25–6.75)
	Lateral Pinch Power (kg)	7.6–9.5	11.00 (10.00–11.50)	10.00 (8.00–11.00)	9.50 (9.25–9.75)	5.50 (5.25–8.25)
	Palmar Pinch Power (kg)	9.3–9.8	10.00 (9.50–10.00)	5.50 (5.00–6.50)	12.00 (10.50–12.00)	5.00 (5.00–7.50)
	Box and Block (numbers)	81–83	67.00 (62.00–69.00)	58.00 (55.00–61.00)	79.00 (71.00–80.50)	62.00 (58.00–65.00)

Values are median (interquartile range). * *p* < 0.05.

## Abbreviations

BG	Basal ganglia
CR	Corona radiata
CRP	C-reactive protein
DBP	Diastolic blood pressure
EPO	Erythropoietin
F-P	Fronto-parietal cortex
G-CSF	Granulocyte-colony stimulating factor
MBI	Modified Barthel index
MMSE	Mini-mental status examination
MRI	Magnetic resonance imaging
RBC	Red blood cell
Reti count	Reticulocyte count
SBP	Systolic blood pressure
WBC	White blood cell
